# The Structure of Clinical Consultation: A Case of Non-Native Speakers of English as Participants

**DOI:** 10.5539/gjhs.v7n1p249

**Published:** 2014-09-25

**Authors:** H. Bagheri, N. A. Ibrahim, H. Habil

**Affiliations:** 1 Faculty of Education, Universiti Teknologi Malaysia, Johor, Malaysia; 2 Language Academy, Universiti Teknologi Malaysia, Johor, Malaysia

**Keywords:** clinical consultation, doctor-patient interaction, multilingualism, multiculturalism, non-native speaker, misunderstanding

## Abstract

**Background:**

In many parts of the world, patients may find it difficult to visit doctors who share the same language and culture due to the intermingling of people and international recruitment of doctors among many other reasons. In these multilingual multicultural settings (MMSs), doctor-patient interactions face new communication challenges. This study aims to identify the structure of clinical consultation and its phases in an MMS where both doctors and patients are non-native speakers (NNSs) of English.

**Method:**

This study takes on a discourse analytic approach to examine the structure of clinical consultation as an activity type. 25 clinical consultation sessions between non-native speakers of English in a public healthcare centre in Malaysia were audio-recorded.

**Findings and Discussion:**

The results show that there are some deviations from the mainstream structure of clinical consultations although, in general, the pattern is compatible with previous studies. Deviations are particularly marked in the opening and closing phases of consultation.

**Conclusion:**

In almost all interactions, there is a straightforward manner of beginning medical consultations. The absence of greetings may have naturally reduced the length of talk. Hence, by directly entering medical talks, the doctors voice their concern on the curing aspects of the consultation rather than its caring facets. The preference of curing priority to caring is more goal-oriented and in alignment with the consultation as an activity type.

## 1. Introduction

In recent years, the intermingling of ethnically diverse societies has been growing faster than ever due to immigration, technological developments, political unrests, tourism, and especially education. These factors have in turn influenced the outlook to multilingualism and multiculturalism in relation to clinical consultations. In such linguistically and culturally mixed communities, people can rarely find doctors with whom they share the same native language and cultural backgrounds (Roberts et al., 2005; Moss and Roberts, 2005). Taking on the assumption that the occurrence of communication problems due to differences in both language and culture in such clinical consultations are higher, it is, therefore, important to investigate how doctors and patients from culturally and linguistically different backgrounds manage clinical interactions.

This paper aims at examining the structure of clinical consultation in a multilingual multicultural setting in which the participants are non-native speakers of English. This study contributes to the extensive literature on doctor-patient interaction by examining the structure of clinical consultations in the context where both participants are non-native speakers of English. Prior studies have predominantly focused on clinical consultations in which at least one of the participants used their native language (Donald, 1986; Ahmad et al., 1989; Roberts et al., 2005; Van Wieringen et al., 2002; Schouten & Meeuwesen, 2006; Meeuwesen et al., 2007; Meeuwesen et al., 2010).

From a pragmatic perspective, rooted in speech act theory (Austin, 1962; Searl, 1969), any utterance has an illocutionary force which is in alignment with the purpose behind its production or perlocutionary force. In other words, the doctor-patient interaction is a goal-oriented type of activity in Levinson’s (1979) perspective with its own constraints. Therefore, the focus of studying the clinical consultation is its purpose including the diagnosis and treatment of disease or delivery of medical services while each turn of interaction is directed towards the involved activity.

As pioneers, Byrne and Long (1976) examined doctor-patient communication in a variety of settings. They described the clinical consultation in terms of a continuum with two extremes. At one end, it is almost doctor-dominated to nearly the exclusion of the patient. At the other extreme, the patient is almost a monologue deciding the content of the talk, the way of presenting the problem, and other details of the talk. They suggest that based on the doctor’s agenda which may be focused on information-gathering or non-directive counseling, the degree of the doctor’s control over the consultation may vary between the two extremes. On the whole, they classified the overall structure of primary care consultations in the following pattern including six phases which has been referred to as the standard structure by most researchers in the following sequential order (Byrne and Long, 1976).

1) Relating to the patient (Opening)
2) Discovering the reason for attendance (History Taking)
3) Conducting a verbal or physical examination or both (Examination)
4) Consideration of patient’s condition (Diagnosis)
5) Detailing of treatment or further investigation (Treatment)
6) Terminating (Ending)

The standard sequential structure of clinical consultation begins with the opening phase which normally consists of an informal conversation with the patient. Generally, the aim of this phase is establishing relationship with the patient in order to start the process of clinical interaction. History-taking is the second phase of consultation during which the doctor attempts to understand the reason for the patient’s attendance by simply asking some relevant questions about the existing health issue. In the third phase, the doctor is involved in examining the patient’s health problem verbally or physically. Diagnosis is the fourth phase in which the doctor presents the patient’s health condition based on elicited medical information. The fifth stage is offering clinical treatment and professional advice by the doctor based on the patient’s diagnostic findings. At the last stage, after making decision on treatment, consultation is terminated.

Since clinical consultation like any other speech event is a structured and goal oriented type of activity (Levinson, 1979) aiming at diagnosis and treatment of the patient (Sarangi and Coulthard, 2000), we start this section by examining the structure of the medical encounters among non-native speakers (NNS). Based on the classic study by Byrne and Long (1976) and the ideal sequence of the structure of clinical consultations by Ten Have (1989), the structure of the data, in this study, is categorized in six phases. Ainsworth-Vaughn (2003) maintains that Byrne and Long’s nomenclature in six phases is based on the doctor’s activity rather than jointly-constructed discourse by both participants. Hence, the role of patients is neglected. To a large extent, this neglect is justifiable because consultation as a speech event is defined and constrained by activity type. During consultation, the doctor is more active and consequently considered more responsible. Responsibility cannot be attributed to an inactive participant. Therefore, the labelling of the stages of activity should logically be based on the more active participant who is the doctor and not anchored on the patient who is the less active participant. We can therefore assign the role of the operator of the ‘machine of conversation’ in Sack’s (1992) terms, to the doctor in clinical consultation. In this way, in addition to controlling interactions, the doctor plays the additional role of a coparticipant. Therefore, it is plausible to attribute the phases of clinical consultation in alignment with the doctor’s activities.

Drew and Heritage (1992) believe that questioning is a crucial discursive feature of organizational interactions. Donald (1997) has investigated three components of information exchange in clinical consultations. The first step is information seeking as the main activity of doctors which is performed around 10 times more than by patients. The next stage is the information-giving turns mostly uttered by patients at the history-taking and symptom presentation. However, doctors normally give information if requested but they rarely present information voluntarily (ibid). Lastly, information confirmation as the third component of information exchange aims to check the effective exchange of information between participants. Next, Heritage and Maynard (2006) explored the complexity of questioning and responding in doctor-patient interactions. This way of examining doctor-patient interaction emphasises information sharing. In fact, this information exchange is a key instrument but not the main purpose of consultation. It does not conclusively consider the goal of the interaction which is the main purpose of consultation. In fact, the participants in clinical consultation do not merely transfer information for its own sake but for the main purpose of diagnosis and treatment of the disease. Since clinical consultation is a goal-oriented activity type with constraints and obligations on participants (Levinson, 1979 and Sarangi, 2010), these features of doctor-patient interaction are not expected to be necessarily compatible with the features of ritual conversation.

In New Zealand as a multicultural society, Gray et al. (2011) concluded that in spite of necessity, doctors do not necessarily use interpreters in their consultations with their patients having limited English proficiency. On the other hand, the literature shows that misunderstandings happen more frequently in consultations with NNSs of English who generally have more limited English repertoire (Roberts et al., 2005). Considering the higher frequency of misunderstandings and communication problems among NNSs, another issue is raised to search to what extent these communication barriers may be evident in or connected to the structure of consultation.

The effectiveness of health messages in terms of three factors including the content of the message, its sources, and the recipients’ typical characters were studied by Tang and Chen (2013). They found that the transmission of the messages aiming at reducing unhealthy weight-control intentions is effective. It indicates that social networking can help the individual avoid adopting risky and unhealthy behaviours.

Considering the trajectory of the goal-oriented interaction, the present study aims to determine how the consultation is structured during the exchange of information and intents between the participants of medical visits. Moreover, the paradigmatic and syntagmatic patterns of consultations are dealt in relation with the construction of consultation. More importantly, this study makes its contributions to this field by searching into the structure of clinical consultation in a new scope where both participants are non-native speakers of English facing more communication barriers to find out whether this change of scope or participants with different language and culture backgrounds has any effect on the pattern of clinical interaction or not; hence, the need for investigating into the matter of interaction more particularly in a new context.

## 2. Method

### 2.1 Participants

A total of four doctors practicing medicine at the health centre in one of the public universities in Malaysia are recruited in this study. The patients are 25 postgraduate foreign students registered at the same university. The doctors aged 35-55 are Malaysians whose first language is Malay while the patients aged 25-50 are native Persian speakers. Because both doctors and patients are non-native speakers (NNSs) of English, they do not share the same first language; hence, English is used as the common language in the consultations.

### 2.2 Procedure

25 audio-recorded consultation sessions between doctors and patients forms the bulk of the data. Data collection was performed in June 2013. The data were also triangulated by observing the consultation sessions and interviewing both groups of participants after the consultations. During the consultations, the researcher was present in the doctor’s room behaving and dressing like a member of medical staff while making observation and taking notes. All the participants were informed about the objectives of the study and confidentiality of the research data. The doctors were approached in the previous day and the patients were invited individually when they arrived at the health centre seeking treatment as a new patient visiting a general practitioner. Finally, both groups of participants were asked to sign an informed consent letter prior to their participation. After data collection, the audio-recorded interactions of the medical visits were transcribed for analysis.

In this pragmatic approach to discourse analysis, the transcribed encounters between doctors and patients as the naturally-occurring interactions were analysed in combination with ethnographic methods of observation and interviews. By doing this, we aimed at the dual purpose of triangulation of the data and increasing the validity and reliability of the study.

First, the recorded data were repeatedly listened and reviewed to identify the content and categorize the general phases of consultation. Then, the recorded data are transcribed using a modified transcription convention after Roberts et al. (2005). Finally, during the analysis of the interaction, whenever needed, the necessary feedback from the participants was taken to assist the robust examination of ambiguous or complicated parts of the data.

Often, at the start of any medical interview, the doctor sets the topic by asking about the patient’s problem. In this way, they contribute jointly to the ‘framing’ of the situation as a clinical consultation. This framing according to Goffman (1986) refers to what is happening in the interaction; the concept of frame makes up the basis of Goffman’s frame theory according to which whatever happens within the context of consultation including any change in ‘frame, footing, and key’ must be interpreted in alignment with the purpose of the activity. In this way, the connection between Goffman’s (1986) frame theory and Levinson’s (1979) conceptual framework as activity type is well established. This study will shed some more light on clinical consultations by discovering the structural features of medical encounters in a cross-cultural setting. Moreover, it has some implication for improving the quality of doctor-patient communications among NNSs.

## 3. Findings and Discussion

Based on the analysis of the medical consultations, the sequential structure of the clinical visits in relation to their contexts and functions is obtained. The following section reviews six different structural phases of the clinical consultations found in this study: opening consultation, history-taking, examination, diagnosis, treatment, and closing.

### 3.1 Opening Consultation

In this study, all 25 consultations begin with very direct, short, and simple utterances which are always pronounced by the doctor aiming at entering context-specific consultation. They are in the forms of questions, requests, orders, opening gambits, gambits and questions, gambits and the patient’s name, pointing to the patient’s seat, and calling the patient’s name preceded or succeeded by questions, requests, or orders. The following are some examples of opening phase.

Data Example 1.1:

1) D: What’s the problem?
2) P: I’ve got cold.
3) D: How long have you got the problem?

Data Example 1.2:

1) D: Ok start it.
2) P: I have a:: one week (.) I have ah: hypertension.
3) D: You were already diagnosed as hypertension?

Data Example 1.3:

1) D: Ok (.) Ah (.) Alright (.) Yes.
2) P: Just a simple cold (.) I think.
3) D: COLD (0.5) How long already?

As examples 1.1-1.3 show, the opening phase of a medical consultation is in concordance with Austin’s (1962) concept of performative speech act. This stage can be considered as a pronouncement by the doctor indicating that they welcome their patients for whom they are ready to perform their professional duties. In almost all interactions, there is a straightforward way of starting medical consultations. The doctor uses body language gestures such as inviting the patient to be seated by pointing to the empty chair, through eye contact, leaning a bit forward especially at the areas head and face, orienting towards the patient to correspond to their body position, and the like. This announcement of performative function of clinical consultation includes an informal conversation aiming at establishing a friendly and confidential relationship with the patient. To this end, the medical consultation is normally preceded by greetings or phatic talk to create a comfortable context for the patient to initiate discussion about the current medical concern (see Byrne and Long, 1976; Ten Have, 1989; Robinson 1998). However, contrary to these studies, there is an absence of any form of greeting or phatic talk in the first stage of consultation. The absence of greeting in the talk may naturally reduce the length of consultation. Moreover, greeting may be dispreferred if it is not consistent with culturally acceptable norms of participants. For example, Moslems do not shake hands with opposite-sex participants or they may prefer ‘salam’ to ‘how are you?’ Hence, by straightforwardly entering medical talk, the doctors voice their concern on curing aspects of consultation rather than its caring facets. It seems that among NNSs of English, medical communication between doctors and patients is mainly medically contextualized and there is very little space for social talk; hence, medical talk is foregrounded at the expense of more socially-bounded interaction.

### 3.2 History-Taking

At this stage, almost every turn uttered by the doctor is either a question or acts as a question as the only way to obtain information about the disease as emotionally experienced by the patient. In addition, any turn by the patient is a response to a question about the problem. To do this, the doctor almost always asks questions followed by the patient’s answers and the doctor’s assessment or feedback. The doctor’s evaluation may not necessarily be pronounced. It may raise another question until coming to an expert diagnosis about the patient’s health problem based on the patient’s self-presentation and lay diagnosis, the information collected by the doctor, and the medically-conducted analysis. The following is a data extract from the history-taking phase.

Data Example 2:

2) Patient: Not really sick but (0.5) actually I have some problem with my skin (lowering his shirt from his shoulders so that the doctor can see the red area which is about 20 by 30 centimeters).
3) Doctor: Ok (1.0) Alright (0.5) Ok (0.5) How long has it been there?
4) Patient: Around 10 years.
5) Doctor: OH (0.5) Long time ago.
6) Patient: Yeah=
7) Doctor: =But is it on and off? Sometimes it comes sometimes it goes? Or is it always there?
8) Patient: No no no (.) just same constant.
9) Doctor: Is it ITCHY?
10) Patient: No.
11) Doctor: Not itchy?
12) Patient: No.
13) Doctor: Just scary?
14) Patient: Yes.
15) Doctor: Does it started with some rash? Started with some WHAT?
16) Patient: No no no (0.5) Nothing just the colour.
17) Doctor: Just the COLOUR (.) Is just there? (0.5) Nowhere else?
18) Patient: No no only here.
19) Doctor: No itchy?
20) Patient: No itchy.
21) Doctor: The problem is just the (.) colour?
22) Patient: Yes yes.

In response to the activity specific question, ‘Are you sick?’ the patient starts displaying his skin problem. Almost all utterances by the doctor are questions or act as questions for the purpose of eliciting information about the patient’s problem. After the patient presents his skin problem in line 2, the doctor’s questions start. In line 3, the doctor uses ‘ok’, ‘alright’, and ‘ok’’ each followed by pauses to allow the patient to say something more or take the turn. When the patient does not elaborate on his problem in the pauses provided for this purpose, the doctor starts questioning on the duration of the problem in the same line. The question ‘How long has it been there?’ indicates that the doctor has got the problem and refers to the problem as ‘it’. The remaining questions are naturally about ‘it’. From the patient response in line 4, after considering the fact that the problem is not new, showing her surprise in line 5, and the patient’s confirmation in line 6, the doctor laches her tripartite question very purposefully in line 7.

The very interesting question in line 7 consists of three inter-related questions. The doctor poses three questions to see whether the skin problems “comes and goes” or “is always there” in three different ways. Since incorrect responses may lead to wrong diagnosis, the doctor simplifies her question repeatedly. The pronoun ‘it’ referring to the disease, is repeated four times, in line 7. This co-referential repetition facilitates understanding and encourages response. Moreover, the word ‘sometimes’ is also repeated to both direct and foreground the patient’s attention towards the frequency of the situation. Lines 9, 11, 13, and 19 are very short and simple questions. However, in lines 7, 15, and 17 all the questions are longer but they are simplified by breaking them into smaller units of enquiries to obtain very specific information. Hence, this simplification helps comprehension of the questions for the patients who are non-native speakers of English.

According to Byrne and Long (1976) the aim of history taking is discovering the patient’s reason for attendance. In doing this, the doctor normally seeks for objective signs while the patient offers the available subjective symptoms as they are perceived. The doctor’s task is to interpret the patient’s subjective talk and reach the accurate objective and professional conclusions.

Analysing the above history-taking sample indicates that questions are the main means through which the doctor can elicit information from the patient regarding their health concerns. Mishler’s (1984) tripartite structure of elicitation structure which includes the doctor’s question, patient’s response, followed by the doctor’s assessment is repeatedly observed. However, each evaluation by the doctor is not uttered to the patient. Instead, each individual assessment triggers a succeeding enquiry in the form of a new question except the last one which is normally pronounced or considered as the diagnosis. During questioning by the doctor, the patient is always very compliant except in the case of non-understanding or misunderstanding which necessitates request for clarification.

### 3.3 Examining

In the continuation of history-taking or verbal examination, the doctor needs to examine the patient’s statements more specifically and through physically observable evidence. These evidential indicators such as physical changes, deformations, pulse rate, body temperature, blood pressure, breathing, etc. trigger at finding some underlying signs related to the patient’s problem. In addition to asking closed questions aiming at more specific information, some on-line and or off-line commentary remarks are presented by the doctor. At this stage the doctor talks more, gives more order, and the patient talks less and is more cooperative in comparison with the two previous phases. The following is an example of examination phase.

Data Example 3:

23) Doctor: Can I have a look again?
24) Patient: (lowers his shirt again to make the affected area visible)
25) Doctor: At the back NO?
26) Patient: Some problems.
27) Doctor: Never been itchy YEAH?
28) Patient: Yeah.
29) Doctor: About 10 YEARS?
30) Patient: Around 10 years.

The doctor at this stage wants to have a second look after history-taking to examine her assumptions in relation with previously collected information. This rechecking is indicated in turn 23 where the doctor requests to look at the affected area for a second time. Again, some questions similar to those of history-taking stage are asked. Questions 25, 27, and 29 are similar to history-taking questions. These questions aim at re-examining some of previously discussed points during history-taking. Usually on-line and or off-line commentaries are associated with the physical examination. At this stage, the search procedure normally ends with a diagnosis or non-diagnosis. Sometimes, there may be a need for further investigations, tests, or referrals to other specialists. In such cases, non-diagnosis becomes as important as diagnosis. At this phase, the objective signs are sought for the purpose of diagnosis.

### 3.4 Diagnosis

In diagnosis phase, after considering the patient’s overall health conditions, the doctor preceded through the patient’s narratives, history-taking, and physical examination. Finally, the doctor announces the findings as diagnostic presentation. At this phase, based on the doctor’s expert knowledge, the search process by the doctor has gone through different stages by which a number of doctor-made hypotheses are checked in consecutive order. Finally, one of them is confirmed after screening different assumptions by means of questions and answers. The last approved hypothesis is normally pronounced by the doctor as the final diagnosis. The following is an example of diagnosis phase.

Data Example 4:

31) Doctor: So there are 2 things since there is not itchy.
32) Patient: Yes.
33) Doctor: One is vitiligo. (1.0) Vitiligo is a eh:: condition in the skin (0.5) Ok. It can become like darken (0.5) because of the melanin (.) Probably melanin but it doesn’t give you any problem (.) It is COSMETICALLY not very nice (.) Another thing it could be a scar from fungi infection (.) May be before you found infection itchiness around here (.) And then for a long time when it gone it will become scary (.) So for the moment what type of cream would you expect to eh::: expect it for me to give?
34) Patient: I don’t know.

In line 31, the doctor introduces her diagnosis on the patient’s problem beginning with ‘so’. This discourse marker indicates that the doctor’s finding is based on rigorous evidence not just a scientific guess. That is, the doctor arrived at this conclusion after considering several possibilities that have emerged from the patient’s self-presentation, the doctor’s oral or physical examinations, and detail analysis from the doctor’s professional knowledge. The longest turn in this session starts in line 33. Immediately after introducing vitiligo as the first possibility, the doctor pauses for a second to observe the patient’s feedback. Not receiving any reaction by the patient, the doctor explains about vitiligo as a skin condition. Similarly, after mentioning the colour change, another 0.5 second pause also triggers the patient’s response but the patient does not use this opportunity to take the turn. During the presentation of diagnosis, the patient listens carefully without any interruption. After attributing the problem to melanin and emphasizing that it is not a medical concern but just a cosmetic one, the doctor then continues to talk about the second possibility. The second possibility is also presented as the scar remaining after recovery from a fungi infection followed by an enquiry about the patient’s expectation regarding the kind of cream to be prescribed.

It is worth mentioning that the patient remains silent during the presentation of diagnosis in an extended turn. The patient does not even give any minimal acknowledgment token or even non-verbal feedback. However, it is to some extent congruent with Heath’s (1992) suggestion indicating that patients normally show minimal acknowledgement tokens during the presentation of diagnosis. During this delivery of diagnosis, the patient even does not minimally react to the first three pauses (i.e. in 1.0, 0.5, and 0.5 sec.) in the beginning of the turn. Despite the doctor’s use of the most extended turn, the patient remains absolutely silent. The patient’s long silence seems logical since the presentation of diagnosis by the doctor at this part of the interaction is totally professional and based on the doctor’s expert knowledge. Therefore, the patient really does not find himself in any type of authoritative position to give any feedback even in case of uncertainty about the doctor’s explanation. Hence, being silent is the best reaction. Likewise, in Iranian culture this type of silence is interpreted as agreement or sign of satisfaction.

### 3.5 Treatment

Treatment is the main purpose for which all other activities are sequentially performed. At this phase, prescribing treatment and medical advice is closely connected to its prerequisite phase which is the diagnosis of the problem. The following is an example of treatment prescription.

Data Example 5:

35) Doctor: Because actually I think vitamin E::: (chiming sound of calling patients to other rooms interrupts the talk) Vitamin E increase regeneration of skin (.) but you take a lo:::ng time (.) It’s not like one month may be it’s three month six month (.) OK.
36) Patient: Thank you.
37) Doctor: It can’t be a big problem.
38) Patient: Thank you so much.
39) Doctor: (typing and speaking loudly) not itching on left shoulder NON ITCHY?
40) Patient: Yes non-itch.
41) Doctor: Actually vitiligo (.) There are no treatment for it (.) but (.) we just give you some vitamin E (.) Ok.
42) Patient: Yes.
43) Doctor: But then this vitamin E (.) They are give may be just (.) enough for two to three weeks.
44) Patient: Two to three weeks (.) Ok I will follow.
45) Doctor: But you will need more.

Normally, immediately after identifying the problem, negotiation for making decision on treatment begins. In this regard, Maynard (1992) suggested the use of ‘perspective display series’ before the delivery of treatment. The doctor, at the end of turn 33, even before finishing her turn on the diagnosis, starts entering negotiation about the treatment by asking about the patient’s expectation regarding the type of cream to be given.

While prescribing treatment, the doctor first draws a causality relationship regarding the role of vitamin E in regeneration of the skin provided it is applied for a long time in line 35. After giving assurance to the patient that vitiligo is not a big problem in turn 37, the doctor rechecks her diagnosis by asking about ‘itchiness’, in turn 39. In turn 41, the lack of treatment for the problem is mentioned and the use of vitamin E and the only choice is reiterated. The treatment advice is followed by emphasizing on the insufficiency of the amount of prescribed vitamin E and need for the continuation of treatment.

The patient’s rejection of the doctor’s invitation to take part in decision-making about the treatment is revealed from the patient’s response as ‘I don’t know’ in line 34. It seems that in the absence of shared knowledge, the patient cannot take part in the prescription of the treatment. In such cases, only enquiry about allergy to some specific drugs seems necessary.

Prior to treatment, doctors may show their inclination, as this doctor does, for shared decision-making if it is applicable. In this regard, the involvement of the participants, contribution of the participants, possession of common information, and arriving at shared concession are of great importance (Charles et al., 1997). However, among NNSs and in MMSs prerequisites for shared decision-making can hardly be achievable due to the language and culture differences.

### 3.6 Closing

Closing clinical consultation is the last phase of the medical encounter as an activity type in which the participants almost conjointly terminate the session. Opening, closing, and turn-taking in conversations are some of the basic notions which were developed by Sacks et al. (1974). These concepts have been widely used in the analysis of interaction since then. At the stage of closing, the participants could review the residue matters, unmet expectations, communication problems, etc. to recapitulate the consultation. Similar to opening phase, the exchange of some social or phatic talk is normally expected to precede the closing or farewell tokens before leaving. However, the following examples illuminate the sudden closing of consultation without any pre-closing remarks.

Data Example 6.1:

46) Patient: Thank you so much. (1.0) Thank you so much=
47) Doctor: = Ok (.) Ehum.

Data Example 6.2:

168) Patient: Thank you.
169) Doctor: Alright.

Data Example 6.3:

42) Patient: Yeah yeah.
43) Doctor: OK (doctor washing her hands and drying them).

In all of the recorded medical encounters, the consultations have abruptly come to a halt as if the ‘turning-taking machine’ of communication in terms of Schegloff and Sacks (1973) has suddenly broken down. Contrary to the expectation, any interaction is brought to a sudden stop without a pre-closing marker or a transitional indicator. Surprisingly and contrary to the expectation, this type of terminating an interaction without any prefacing or discourse marker is very anomalous. This way of terminating interaction is incompatible with Schegloff and Sacks’ (1973) view who believe that the ‘turn taking machine’ should be brought to a stop after the fulfillment of its purposes. Accordingly, an interaction is not expected to stop by itself without signaling the completion of its function in an appropriately acceptable manner.

The absence of phatic talk or rather the contraction of phatic talk to a mere greeting may be a sign of the time constraints faced by doctors in many public health centers. This may be a time saving strategy to decrease the length of clinical consultations. The data showed that the sessions are between 1:39- 12:41 minutes long with the average length of 5:30 minutes per visit. Focusing on the medical talk can therefore reduce the length of consultation by trimming some peripheral parts from both the beginning and the end of each clinical session.

At the stage of opening, the announcement of the co-participants’ readiness to start the activity of consultation which is already arranged through the processes of securing an appointment, obtaining a queue number, and scanning the queue number outside the doctor’s examination room, prior to being called in by the doctor, seem to be necessary and consistent with the type of involved activity. Hence, the direct way of opening the consultation by the doctor foregrounds the curing activities for which they have been trained to do the expense of caring undertakings about which they do not normally have enough experience especially in dealing with people from different cultures. Moreover, time shortage and institutional obligations can also discourage the doctors from attending to the socio-cultural aspects of the patient’s concerns. Likewise, Heritage (1977) attributes narrowing and respecifications of options in conversations to institutional discourse causing restrictions on speech activity. This focus on core medical activity by direct involvement to ask about the reason for attendance seems to be a kind of restriction which is institutionally imposed on interaction among non-native speakers who may not have a clear cultural understanding of each other.

From this perspective, the absence of social talk in consultation seems consistent with Ai’s (2013) findings discovering that the computer and internet application negatively affect the user’s mental health in Korea by lowering initial friendship-closeness. Since Korea has a high-context culture similar to that of the participants of this study, the doctor’s frequent involvement with the computer and internet may have affected their behavior in medical consultations resulting in concentration on the core activities of consultation (phases 2-5) and disregarding its social aspects (phases 1 and 6). This dichotomy of the voices was already established by Mishler (1984). Moreover, it is the voice of medicine which is applied in the core medical activities and later is reported in the patient’s electronic file rather than the world-life voice which makes up the beginning and end of medical conversations. Therefore, the doctor is institutionally obliged to focus more on what is recorded than what is heard and will later vanish in the air.

Similarly, the intervention of computer technology in doctor-patient communication, especially its supervisory effects may add to the doctor’s stress similar to the effect of a third participant during a medical visit. In such a condition, the doctor is obliged to not only act to satisfy the patient, but also to interact with the machine which may be more difficult and whose recorded files may be accessible to other colleagues and the health system authorities. In this way, it is also prone to criticism.

Another worth-mentioning finding of this research which looks different from the previous studies pertains to the way the doctor presents the diagnosis. In doing this, except in cases where the patient’s lay diagnoses are approved or it is already known to both, the doctor explicates the identified problem in an extended turn. During this presentation, the patient remains silent, without showing any acknowledgement – almost oblivious to the doctor’s ‘self-talk’. This is in contrast to a previous study which indicates that patients normally show minimal acknowledgement tokens during the presentation of diagnosis (Heath, 1992).

Generally, healthcare providers have difficult task of making a compromise between the institution demands and the patients’ affective needs. That is, the former necessitates task completion while the latter gives priority to compassion (Ruusuvuori, 2007). This can be another reason behind the greater focus on medical voice and mitigation of social talk. More importantly, in an MMS, providing such a balance of needs is a big challenge because of language and cultural differences when the talk itself is a problem (Roberts et al., 2005; Moss and Roberts 2005).

This study supports the findings of another study on clinical consultations which are not satisfactory from the patients’ perspectives in Indonesia (Claramita et al., 2011). In this research, the doctors themselves declare that they did not intentionally practice the current paternalistic and one-way style of communication with their patients. The researchers attribute the problems to the culture and propose the application of partnership in doctor-patient communication in Southeast Asian context.

Bagheri, Ibrahim, and Habil (2012) concluded that in addition to time constraint, the complexity of dealing with phatic talk in the first and last phases of clinical consultation may have contributed to the shortening of clinical consultation in addition to decreasing the possibility of communication problems in the initial and final stages of medical encounters in the MMS under the study. Accordingly, the occurrence of misunderstanding is mainly searchable in the history-taking, examination, diagnosis, and treatment phases which are the coral stages of the consultation.

The presence of patients’ companions could have influenced the structure of clinical consultation (Robson, Drew, & Reuber, 2013). They have found that in spite of equal duration between dyadic and triadic consultations, in interactions in which the patient is accompanied by some companion, the patient speaks less than in unaccompanied visits. This idea is further supported by Berger (2012) who emphasizes improving the quality of medical visits by doctors and disproves the referral of difficult patients to a bioethics consultant as an inefficient solution.

Taking into consideration language and cultural aspects which structure the clinical consultation among NNSs, the application of the internet for the purpose of health enhancement is approved by Baek and Yu (2009) in a cross-cultural context. Similarly, Tang & Chen (2013) confirmed the application of social networking as an effective means of communicating health messages leading to healthy behaviors.

## 4. Conclusion

From the findings of this study we can conclude that the participants seem to be focusing more on the core medical phases of the consultation including history-taking, examining, diagnosing, and treating, to the expense of non-medical or socio-cultural talks in the beginning or end of consultations including opening and closing phases. This pattern of consultation is in alignment with Mishler’s (1984) dichotomous distinction between the ‘voice of medicine’ and the ‘voice of the lifeworld’. That is to say, from the very beginning stage of the consultation, the use of socio-cultural discourse is suppressed in favour of the use of voice of biomedical discourse even at its most appropriate context and prior to entering the core medical topic of the patient’s attendance.

In this study, the absence of ritual talk in the opening and closing stages of medical interaction appears to be in agreement with Roberts et al. (2004). In their study with non-English speaking patient, they found that openings are frequently protracted and appear to be more difficult part of interaction for both participants. Likewise the doctors in this study keep their phatic communication with the patient as simple and concise as possible to decrease the contingency of communication problems.

Misunderstandings in medical communication can be more possibly interpreted as medical errors or mistakes; hence, more prone to lack of understanding by the patient. However, misreading of socio-cultural talk may be more face-threatening with its detrimental effects. Consequently, circumvention or avoidance of phatic talk may decrease the possibility of social misunderstanding.

In consultation among NNSs as an activity type reflecting the conceptual framework of the study (Levinson, 1979; Sarangi, 2010), interaction can be affected by the intrinsic motivation of the participants as indispensible determinants of the goal of medical visits. From the doctor’s perspective, the delivery of quality health care has priority to be considered as the main goal. On the other hand, from the patient’s point of view, the existing medical concern is of the highest priority for which a medical visit is arranged. Therefore, both interactants are eager to conjointly focus their attempt on curing rather than on caring aspects of the consultations.

In sum, all the encounters have been in progress routinely in alignment with the purpose of each consultation session. Likewise, the process of interactions continued according to the sequential pattern of clinical consultation in six phases starting with opening and ending in closing stage. However, the absence of ritual talks in the opening and closing phases of the consultations are the significant differences discovered in this study. These types of sudden beginning and ending clinical consultation are incompatible with different findings in the literature. The presentation of diagnosis in an extended turn by the doctor without any observable reaction by the patient was another new finding of this research. The structure of the consultation in the MMS under study among NNSs of English is such a purely goal-oriented activity which eliminates the non-medical or social talks. Hence, the voice of medicine dominates the medical visit.

As a final remark, it is worth mentioning that this study was conducted in a government clinic where the patients receive free medical treatment. In addition, the participants come from different culture and language backgrounds. Hence, the findings are not applicable to private settings or to first language contexts.
